# Feedback in radiology: Essential tool for improving user experience and providing value-based care

**DOI:** 10.1186/s13244-025-02002-9

**Published:** 2025-06-26

**Authors:** Andrea Rockall, Jacob J. Visser, Cristina Garcia-Villar, Naama Lev-Cohain, Patrick Omoumi, Marie-Pierre Revel, Ruth Mary Strudwick, European Society of Radiology (ESR), on behalf of the ESR and ESR Value-based Radiology Subcommittee, Judy Birch, Boris Brkljačić, Philip Robinson

**Affiliations:** 1 Department of Surgery and Cancer, Faculty of Medicine, Imperial College London, London, UK; 2 Department of Radiology, Imperial College Healthcare NHS Trust, London, UK; 3 Department of Radiology & Nuclear Medicine, Erasmus MC, Rotterdam, The Netherlands; 4 Hospital Universitario Puerta del Mar, Cádiz, Spain; 5 Department of Radiology, The Faculty of Medicine, Hadassah Medical Center, Hebrew University of Jerusalem, Jerusalem, Israel; 6 Department of radiology, Lausanne University Hospital and University of Lausanne, Lausanne, Switzerland; 7 Université Paris Cité, Paris, France; 8 Assistance Publique des Hopitaux de Paris, Hôpital Cochin, Paris, France; 9 University of Suffolk, Neptune Quay, Suffolk, UK; 10 Am Gestade 1, Vienna, Austria; 11 Pelvic Pain Support Network, Poole, UK; 12 Department of Diagnostic and Interventional Radiology, University Hospital Dubrava, University of Zagreb School of Medicine, Zagreb, Croatia; 13 Leeds MSK Radiology, Leeds, UK

**Keywords:** Value-based health care, Feedback, Patients, Referrers, Hospital management

## Abstract

**Abstract:**

Measuring the value that radiology brings to patient care can be challenging. A positive patient experience is consistently associated with patient safety, clinical effectiveness, and outcome measures and is therefore a tool for measuring value-based care. Monitoring the experience of users of radiology services is an indispensable component of quality improvement programmes for radiology departments. The integration of comprehensive feedback mechanisms brings numerous benefits, including enhanced care, strengthened trust, and greater engagement with our stakeholders and service users. Feedback should be collected from a variety of stakeholders through a 360-degree approach, combining both systematically performed structured methods, such as formal surveys, and unstructured methods, such as informal and opportunistic information gathering during multidisciplinary rounds. To maximise the impact of feedback, it should be frequent and diverse, ensuring that all perspectives are considered. Leaders in radiology must prioritise embedding a culture of feedback within their institutions, recognising its crucial role in continuous improvement. It is essential to ensure that our departments consistently provide value to our most important stakeholders—the patients—but also to our referrers and trainees. In this article, we consider methods for collecting feedback and provide some of the key findings from the literature. By fostering an environment that values and acts upon feedback, we can achieve significant advancements in patient care and overall service quality in radiology.

**Critical relevance statement:**

Regular feedback from patients, peers, radiographers, referrers, trainees and other users of imaging services is an essential tool for continuous quality improvement, patient safety and value-based care, enhancing trust and greater engagement with our stakeholders and service users.

**Key Points:**

- Feedback from patients and referrers, radiographers and radiology trainees, helps radiology departments to identify weaknesses and strengths, and should be fully incorporated into daily practice.
- Many methods are available for collecting user and stakeholder experience, and these should be implemented as a priority.
- Acting on stakeholder feedback can improve patient safety and patient experience ratings, leading to a culture of continuous improvement in value-based care.

## Introduction

The value that radiology brings to patient care can be difficult to measure. Staff working in radiology departments interact with a wide group of stakeholders, including patients and carers, referring colleagues, trainees, and hospital management. User-experience feedback is one tool we can use to measure our value. Patient experience in imaging commences once a referral is made to the radiology department and encompasses events at the time of booking, the appointment, and through to the point of receiving results. The entire team in the department is involved in ensuring an excellent patient experience, and this must be recognised when training staff at all levels. Evidence suggests that effective communication between radiologists and patients benefits both parties, improving safety, satisfaction, and health outcomes for patients and benefits radiologists in multiple ways at personal, clinical, and professional levels [1]. In a systematic review, patient experience was found to be consistently positively associated with patient safety and clinical effectiveness across a wide range of disease settings and outcome measures [2].

Monitoring patient experience is an essential tool for quality improvement in radiology departments, helping to identify areas that provide an appropriate experience for patients, as well as highlighting areas for improvement. Matching the expectations of a wide variety of patients can be challenging, particularly in resource-limited healthcare settings, but starting with simple areas for improvement can make a significant difference to patients (Table 1).

**Table 1:** Selection of publications that focus on patient experience and feedback in radiology

First author and year	Type of publication	Key objectives	Key findings	Conclusions
Review articles on patient-centred care and patient experience				
Brook 2017	Review article.	To explore factors that impact patient perception and experience with imaging services.	Authors present different ways to collect patient feedback, with pros and cons of different methods. Typical feedback items and reasons for complaint are presented.	Collecting feedback is important, but analysis must lead to identification of improvements to be made, implemented and monitored.
Narayan 2019	Expert panel narrative review.	Patient-centred care, health equity, with concrete examples of how to foster patient-centred, equitable care in radiology.		
Schreyer 2022	Narrative review.	Medline search for patient-centred radiology.	Authors identified that most published literature is based on surveys evaluating the current status, with few studies focusing on interventions to improve patient experience.	Value-based radiology requires a focus on optimising patient communication.
Original articles on patient experience or multisource feedback				
Kapoor 2019	Impact of a Multifaceted Intervention on National Ranking.	Initiated patient experience survey and undertook quality improvement exercise during the study period.	Found an increase in national ranking from 35th to 50th percentile	The electronic patient experience survey identified areas for improvement that led to a measurable improvement in the national rank.
Van den Berg 2019	Patient complaints in radiology: 9-year experience at a European tertiary care centre.	To collate the number of complaints over time and the main nature of complaints.	Authors found 14.4 written patient complaints per 100,000 radiology procedures. The most frequent written complaints were related to interventional procedures. Main complaint categories were quality (34%), safety (22.3%), timing and access (181%) and communication (18.1%).	Knowledge of the reasons for complaints may reduce their number and improve patient care.
Curran 2022	Evaluation of a multisource feedback tool and peer coaching.	Pilot 360-degree MSF assessment and coaching to identify impact on reflective practice.	Respondents found improvements in self-directed learning and reflection on practice, affirming what they were doing well and identifying opportunities for professional development.	MSF and peer coaching were found to be key elements in enabling reflection on own practice to consider strengths and weaknesses, to strengthen professional development.
Kapoor 2022	Evaluation of the impact of online posting of patient experience scores for radiologists.	To compare patient experience scores between radiologists and non-radiologist physicians and to assess changes in scores after their public posting in an online physician directory.	Ratings were slightly lower for radiologists compared to non-radiologists, except when the radiologist undertook an invasive procedure, when scores were similar to non-radiologists. Ratings increased over the study period. For radiologists, the largest improvement for related to the provision of post-procedural follow-up care instructions.	The study findings support the implementation of patient experience surveys in radiology. Increase in ratings over the study period may be due to the online posting of ratings.
Abuzaid 2023	Patient survey on patient experience and satisfaction with imaging services.	A descriptive cross-sectional survey to obtain insights into patient experience, staff attitudes and overall satisfaction.	Over 400 participants completed the survey. Authors found that waiting times and staff attitudes significantly affect patient satisfaction.	Imaging departments should prioritise efficient appointment scheduling, reduced waiting times and foster positive staff-patient interactions. An open-ended feedback form allowed for valuable suggestions from patients.
Doshi 2016	Evaluation of an online ratings website.	To use patient reviews to identify factors associated with positive and negative patient perceptions of imaging centres.	Eighty-six per cent of comments related to service quality, with only 14% related to the radiologist, commonly around professionalism (positive or negative). Perceptions were largely shaped by service quality, heavily influenced by schedulers, receptionists, and technologists.	Radiology departments need to adopt a service-oriented.
Evaluations of written radiology reports				
Heye 2018	Survey of Swiss GPs and hospital referrers on style of imaging reports.	To assess the perception, preferences and expectations of recipients of radiology reports.	Over 500 respondents Structured text and images were rated highest with regards to readability, time and good communication. Eighty per cent stated that the report should allow fast and efficient reading.	Valuable user feedback allows the radiologist to understand the needs of the referrer. Found a strong argument in favour of structured reporting.
Kemp 2022	Evaluation of the use of patient-friendly reports on the patient portal.	Patient-friendly reports were made available in a private practice setting, and statistics of use were collected.	Fifty-nine per cent of patients accessed their reports. Those who reviewed the patient-friendly report, spent an average of 5.8 min compared to the raw report, 1.8 min. Over 80% of patients reported that diagrams and definitions helped them understand the report.	Authors concluded that implementation was feasible and increased patient engagement and subjective patient understanding, with a positive impact on patient experience.
Vincoff 2022	Review article of patient-friendly radiology reporting.	Review of the role of the radiologist report within the context of patient and family centred care, review current strategies and investigations in patient-friendly reporting, and summarise radiology reporting challenges and opportunities for the future.	Several different methods have been employed to improve the patient-friendly nature of radiology reports, including summary statements, structured reports, embedded videos and diagrams and text translation to lay language.	Patient-friendly reports offer significant opportunities for radiologists to improve the quality of care.

It is equally important to listen to the experience of radiographers and our referring clinicians. This includes evaluating ease of referral, efficiency of appointment scheduling, turnaround time for receiving results, accessibility of radiologists for consultations, and the clarity and utility of our reports. The latter is particularly true with complex cases where there may be a risk of misunderstandings if the report is not clear, with the potential for incorrect transmission of the imaging interpretation to the patient. Additionally, their preference for the form of our reports (structured vs unstructured) may also differ. It is not always possible to meet all the expectations of our referrers, but being open to continuous improvement is important for constructive and safe working environments.

Interactions with radiographers, referring clinicians, multidisciplinary team meetings, and patient-reported outcomes are also important sources of feedback, which can be leveraged by radiologists to continually improve their diagnostic accuracy and overall contribution to patient care.

Gathering this multisource feedback allows shaping the strategies for change and improvement. In this article, we consider the key goals and potential benefits of obtaining patient feedback, as well as feedback from the wider healthcare community. In addition, we review the different methods and results of obtaining feedback in radiology, and the potential for quality improvement and value-based care.

## Feedback from patients as users

As imaging professionals, we should provide compassionate and sensitive care to make the entire patient experience in our departments as positive as the circumstances allow. Asking our patients and learning about their experience and values is essential, to understand what is important to them, ensuring patient-centred care [3, 4]. Otherwise, we can easily make assumptions and think that we know what a patient might want from our service. Understanding what is important to the patient is part of values-based practice (VBP), which is the consideration of a patient’s values in decision-making. By patient’s values, we mean the unique preferences, concerns, and expectations that each patient brings to a practice encounter, including imaging. These unique values must be integrated into any decisions about the care of the patient [5].

Staff working in the radiology department can do all of this by giving the patient the opportunity to explain what is important to them and by providing the patient with enough information so that they can make an informed choice. No decisions should be made about the patient without their involvement: ‘No decision about me without me’ [6].

Patient feedback can help staff in the radiology department identify areas in which they are doing well and areas that need improvement, embracing a culture of openness, listening to and learning from criticism. Feedback may identify blind spots that staff were not aware of. Patients need to know that their voices are being heard, instilling a culture of kindness. This can lead to clear benefits to patient care as staff can act on the feedback and become more aware of what is important to their patients, leading to improved patient satisfaction (Table 1). Good feedback from patients can help to motivate staff, create a culture of continuous improvement and increase job satisfaction.

In some countries where patients select a healthcare facility based on the rankings of the department or the physician, having patient feedback and being able to demonstrate actions as a result of this may improve the ranking of the department [7]. However, there is limited information concerning the extent to which radiology departments in Europe gather patient feedback, and this is an area for further research through the European Society of Radiology.

## What methods are currently in use, and results of studies on patient feedback?

Establishing strategies to obtain patient feedback directed at the imaging department is essential. In the United States, under the Hospital Value-Based Purchasing Programme of the Centres for Medicare & Medicaid Services, patient satisfaction accounts for 30% of the measures of and payments for quality of care [8]. Although this is unique to one country, it is recognised that quality metrics are directly related to patient experience, as well as patient outcomes [9].

Feedback can be gathered both in a structured manner, such as through the implementation of formal surveys, and in an unstructured manner, like verbal feedback and informal opportunistic information gathering during multidisciplinary rounds. Various methods are currently in use for collecting patient feedback at the time of attendance or post-visit, and it is important that departments offer a variety of options, including internal surveys that could be verbal, paper-based or digital formats, such as touch screens or on mobile devices [9].

Internally designed surveys can be tailored to the specific needs of the practice and to previously identified areas of improvement, and offer room for free comments [10, 11]. Successful surveys need to consider the patient, for example, a long survey could be difficult to complete [12], or older patients could prefer a paper survey, whereas the younger patients would prefer an electronic survey [13]. Additionally, surveys should be anonymised and solicited shortly after the interaction, ideally as a mandatory offer following each imaging interaction. Patient advisory groups may be a resource for developing feedback questions, to identify areas that patients would like to feedback on, for example, whether they received a copy of their report, whether they perceived the staff as being kind and empathic and whether the next steps were clear (survey questions are available as Supplementary material in [14]).

Some initiatives allow reviews to be posted online by both referrers and patients, similar to Trust Pilot or Amazon reviews, by providing a satisfaction survey at the end of each report [10, 15–17]. In one such site, approximately 75% of the feedback is from patients, and a common complaint was the incomprehensible nature of the radiology report [18]. This feedback is important for us to understand patients’ needs and think about how these needs can be met.

How do we interpret the results of patient feedback? Bad feedback is not always due to a bad doctor or a bad department. Feedback to radiology departments may, at times, reflect problems encountered outside of the radiology department and isolating radiology from the whole care pathway can be a challenge. Disgruntled or upset patients may at times give highly critical feedback—analysis of over-arching trends, as well as paying attention to individual complaints, needs to be balanced. However, openness to reflection on poor feedback is one of the first steps that allows positive change, as was found in this study that reviewed patient complaints over a 9-year period [19].

Human factors impact how patients perceive radiologists, affecting the perception of quality of care. The website RateMDs found the most frequent score was ‘excellent’ (63–74%), followed by ‘terrible’ (14–20%). Positive reviews included the words caring, knowledgeable, and professional, whereas negative reviews included the words rude, painful, and unprofessional [20]. Open discussion to find ways to improve feedback can be the focus of department away-days or retreats, or within departmental meetings.

## Feedback from our referrers and the impact of our reports on patient care

Effective communication between the referring physician and the radiologist is essential for optimising the choice of imaging modality and ensuring the accuracy of the final radiological report. This directly impacts patient treatment and influences the economic aspects related to the costs of these exams [21].

Reports are the radiologists’ key means of communication with referrers and patients, and feedback is essential to meet their expectations and ensure that our reports are actionable, ensuring value to patient care. Reports need to be clear and comprehensible, and answer the clinical question being posed as best as possible.

Conducting surveys using structured questionnaires is the most immediate way to obtain feedback from the referring physicians, as well as patients. Surveys have highlighted a range of concerns, including simple typos, lack of templates, accurate addressing of the clinical problem, and the urgent need for personal communication of report results to the referring physician [22–24].

In an emergency department setting at a university hospital, a 9-question online survey was conducted among 68 healthcare professionals. The main recommendation was to standardise report structure, style and lexicon, in addition to being focused, timely and brief. In this context, a long list of possible differentials was deemed irrelevant [25].

In a non-emergency setting, expectations may be different. Regarding the need for a focused radiology report, another study showed that 63% of clinicians agreed with the statement that “not mentioning an organ or body part in a radiology report implies that the radiologist has not examined it closely” [26].

Conversely, radiologists frequently mentioned the lack of accurate clinical information provided with the imaging request, negatively affecting their interpretation and potentially harming patient care [27–30]. Poor communication between referrers and radiologists has the potential to result in inappropriate radiology requests for the wrong procedure, and a lack of correct referral information may lead to errors or inaccuracies in the radiology interpretation, potentially leading to incorrect patient management with resultant potential harms and inefficiencies. These issues may result in a lack of confidence in the radiology report and underutilisation of its recommendations; good feedback mechanisms between referrers and radiologists could enhance appropriate use and avoid errors.

A major potential solution is to increase both personal communication, whether by phone or face-to-face, and the use of peer-review systems between radiologists and clinicians [22, 24, 29]. The advantage of personal communication is evident in multidisciplinary meetings, where the radiologist plays a crucial role in joint decision-making and participates in clinical discussions, as well as final recommendations [31].

The implementation of structured feedback systems between clinicians and radiologists could potentially address these concerns. This iterative feedback loop could evaluate both the written radiology report and the adequacy of the clinical information provided by the referring physician. Although time-consuming for all participants, it is bound to improve the process for everyone involved [21, 29, 32].

The increasing participation of radiologists in the multidisciplinary committees brings us important and often immediate feedback concerning the patient’s care through discussions with other clinical colleagues. This scenario allows us to discuss the best diagnostic-therapeutic option for each patient and to establish new management protocols. Reviewing specific cases and subsequently detecting some radiological mistakes can improve our practice.

Radiologists’ reports, like many other medical report components, are increasingly accessible to patients via web-based portals, allowing direct written communication between the radiologist and the patient. The portal method was preferred over other ways of communication by 79% of patients questioned in a survey involving 53 participants [33]. However, the preference for having immediate access to reports depended on the scenario. It was requested by 60% of participants in case of nearly normal results, and by only 47% in case of serious abnormalities. Indeed, direct patient access to radiology reports raises a number of questions, including what content to include and whether there should be an embargo period to allow communication with the treating physician, though this may be regulated at a national level. A period of embargo may be particularly crucial in the case of stressful content, such as the description of disease progression in oncology patients.

It is also very important to evaluate what patients understand about their radiological reports.

In a study randomly selecting 8 reports from PACS, which were evaluated by a total of 104 patients, the presence of unclear technical language was mentioned in 60% of the evaluations. The most common finding expressed in 20% of the evaluation forms was a request for an explanation of the report in lay terms [34]. In some cases, direct verbal communication is the ideal mode of communication, as radiologists are in the best position to explain complex imaging results. In one study, a large percentage of patients (64%) responded positively to wanting to meet the radiologists interpreting their exams [35].

Although structured reporting improves clinician satisfaction [36], interpretability remains limited for patients [37]. This is important feedback for the radiology community, and innovations that allow rapid ‘translation’ of radiology reports to lay language, or indeed mini explanatory videos, are areas of research interest [38]. A good understanding on the part of the patient is essential for shared decision-making and a positive impact on patient experience [39, 40].

The impact of the radiologist’s report on patient care is obvious in the case of unsuspected findings, such as the discovery of an incidental pulmonary embolism during oncology follow-up, requiring urgent treatment [41]. The impact on patient care is also significant for other cases of unsuspected diagnoses, such as the incidental discovery of a renal tumour [42]. Radiologists are increasingly required to take part in multidisciplinary meetings, contributing to treatment planning, beyond the context of oncology tumour boards, and this trend reflects the value of imaging in the patient care pathway [43]. Radiologist participation in multidisciplinary teams (MDTs) improves reflective practice, decision-making and prevents the likelihood of isolation. Radiologists’ confidence in their clinical decision-making increased when there was immediate feedback from pathologists [44].

## Feedback from our radiographers, radiology residents, and other radiology colleagues

Feedback from radiographers and radiology colleagues, and residents is essential for enhancing clinical practice and professional growth in radiology [45, 46]. Peer review processes are a cornerstone in this feedback system, ensuring that diagnostic quality and accuracy meet the high standards required in medical practice [47]. Peer review allows radiologists to receive constructive criticism on their imaging interpretations, identifying areas for improvement and reinforcing best practices. This collaborative approach fosters a culture of continuous learning and excellence within radiology departments. An important tool can be a regular meeting to anonymously review errors and misses, in a non-confrontational and anonymous format, with the value that the department can learn from these mistakes and find ways to reduce errors. It can be motivating to also include good spots or difficult diagnoses that were correctly made, as a positive side to the meeting [48].

For residents, the benefits are substantial as they gain insights from experienced radiologists, helping to refine their diagnostic skills and clinical judgement. Regular feedback mechanisms such as case discussions, morbidity and mortality meetings, and structured reporting audits provide opportunities for residents to learn from both successes and mistakes under the guidance of their mentors. Teaching in the workplace has been identified as being protective against burnout, and constructive feedback sessions can be a component of teaching sessions [49].

Structured feedback systems, including formal evaluations and anonymous surveys, help gather comprehensive and honest feedback from peers. Studies have shown that peer review in radiology can significantly improve diagnostic accuracy and professional development, highlighting its importance in maintaining high standards of care [2].

Appropriate software can integrate peer review and structured feedback into daily clinical practice [50]. Radiology-specific platforms facilitate efficient review and feedback processes, enabling radiologists to share and analyse images, provide annotations, and track performance metrics. These tools support collaborative learning by allowing for the easy dissemination of feedback and educational materials. These software solutions also offer analytics capabilities, helping radiologists identify patterns and trends in diagnostic errors. This data-driven approach enables targeted interventions and continuous improvement. For instance, a tool like RadPeer streamlines peer review workflows, ensuring timely and structured feedback [51].

## Focus on feedback from the hospital management team: leveraging feedback to help improve imaging services

Radiology departments should design some communication strategies not only with patients and other clinical colleagues, but also with the hospital management, to align with the institutional strategies. It is important that this is an open two-way conversation. Institution-wide yearly staff surveys can test the mood of employees and identify areas of concern that may be addressed where feasible. This type of activity is critical in this time of severe workforce shortage, in radiology, as well as in healthcare generally. Supporting the workforce post-pandemic has been critical in order to retain highly trained staff.

However, one key role of the feedback received from patients and referrers is to influence and leverage requests for improving infrastructure and resources in the radiology department. The need for supporting additional space and equipment for the department may be supported by the evidence obtained from referrer and patient feedback, if they experience unacceptable delays for diagnostic tests or results turnaround times. This evidence can be a powerful tool to justify additional support. This can be supported by internal strategies to measure departmental workflow and efficiency, together with establishing quality and quantity indicators.

The extent to which feedback systems have been implemented in radiology departments across Europe is not known, and the barriers to implementation are also not widely understood. It is possible that workforce shortages, in addition to a fear of negative feedback, may have contributed to the slow implementation of feedback systems. However, the benefits that can be obtained through feedback need to be emphasised in order to strengthen this area of radiology practice.

## Conclusions

Measuring the experience of users has become an indispensable component of quality improvement programmes for radiology departments. The integration of comprehensive feedback mechanisms brings numerous benefits, including enhanced care, strengthened trust, and greater engagement with our stakeholders and service users. Feedback should be collected from a variety of stakeholders through a 360-degree approach, combining both systematically performed structured methods, like formal surveys, and unstructured methods, such as informal and opportunistic information gathering during multidisciplinary rounds.

To maximise the impact of feedback, it should be frequent and diverse, ensuring that all perspectives are considered. Leaders in radiology must prioritise embedding a culture of feedback within their institutions, recognising its crucial role in continuous improvement. It is essential to ensure that our departments consistently provide value to our most important stakeholders—the patients—but also to our referrers and trainees. By fostering an environment that values and acts upon feedback, we can achieve significant advancements in patient care and overall service quality in radiology.

As we move forward, the ESR value-based radiology (VBR) subcommittee can play a pivotal role in developing tools and strategies to support the integration of feedback systems in radiology departments and consider what actions should be considered as most effective. Providing practical examples, guidelines, and resources will help departments implement and sustain these feedback mechanisms. Ultimately, by prioritising and acting on feedback, we can ensure that our radiology services meet the highest standards of care and continue to improve in response to the needs of our patients and the healthcare community.

## Abbreviations

MDTs: Multidisciplinary teams
VBR: Value-based radiology
VBP: Values-based practice
